# Overexpression of *HVA1* Enhances Drought and Heat Stress Tolerance in *Triticum aestivum* Doubled Haploid Plants

**DOI:** 10.3390/cells11050912

**Published:** 2022-03-07

**Authors:** Harsha Samtani, Aishwarye Sharma, Paramjit Khurana

**Affiliations:** Department of Plant Molecular Biology, University of Delhi South Campus, Benito Juarez Road, New Delhi 110021, India; harshasimply.sam@gmail.com (H.S.); aishwaryesharma91@gmail.com (A.S.)

**Keywords:** ABA, doubled haploid, drought, heat, HVA1, LEA, stress, tolerance

## Abstract

Plant responses to multiple environmental stresses include various signaling pathways that allow plant acclimation and survival. Amongst different stresses, drought and heat stress severely affect growth and productivity of wheat. HVA1, a member of the group 3 LEA protein, has been well known to provide protection against drought stress. However, its mechanism of action and its role in other stresses such as heat remain unexplored. In this study, doubled haploid (DH) wheat plants overexpressing the *HVA1* gene were analyzed and found to be both drought-and heat stress-tolerant. The transcriptome analysis revealed the upregulation of transcription factors such as *DREB* and *HsfA6* under drought and heat stress, respectively, which contribute toward the tolerance mechanism. Particularly under heat stress conditions, the transgenic plants had a lower oxidative load and showed enhanced yield. The overexpression lines were found to be ABA-sensitive, therefore suggesting the role of *HsfA6* in providing heat tolerance via the ABA-mediated pathway. Thus, apart from its known involvement in drought stress, this study highlights the potential role of *HVA1* in the heat stress signaling pathway. This can further facilitate the engineering of multiple stress tolerance in crop plants, such as wheat.

## 1. Introduction

Different abiotic stresses such as drought, salinity, cold, and heat are the major factors that affect plant growth and development. In response to these stresses, plants have evolved defense mechanisms that consist of proteins that directly or indirectly aid in abiotic stress tolerance. The late embryogenesis abundant (LEA) protein belongs to one such family of proteins that decrease the cell damage and protect the cells under abiotic stress conditions [1,2]. The isolation of the first LEA protein was achieved from cotyledons of cotton and, as it accumulated in the late embryonic stage, it was named as LEA [3]. Thereafter, LEAs have been found to express not only during the late stage of seed maturation but also in various vegetative organs such as root, stem, leaves, and other tissues throughout the plant development [4,5]. Moreover, LEA proteins have been reported in different organisms such as *Cyanobacteria*, *Arabidopsis thaliana*, *Oryza sativa*, and *Triticum aestivum* and in prokaryotes such as Rotifers, which highlights their wide distribution [1,6].

On the basis of conserved motifs, amino acid sequences, and the phylogenetic relationship, LEA proteins in plants have been classified into eight groups: LEA1, LEA2, LEA3, LEA4, LEA5, LEA6, dehydrin (DHN), and seed maturation protein (SMP) [7]. Most LEA proteins range from 10 to 30 kDa and are mainly composed of a repeated arrangement of hydrophilic amino acids that lead to the formation of highly hydrophilic structures [8]. As they have a high net charge and low hydrophobicity, this allows them to function as molecular chaperones and help prevent the formation of damaging protein aggregates during water stress [7,9]. LEAs belonging to each group have a unique conserved motif that is preserved through the course of evolution and are essential for their protective role(s) under different abiotic stresses [1,10].

Different members of LEA appear to localize in different cellular compartments such as the cytosol, nucleus, mitochondria, endoplasmic reticulum, and chloroplast. This highlights that every compartment of the cell requires LEA to provide protection against the stress conditions [11]. LEAs have been found to be expressed under various abiotic stresses such as drought, heat, and cold [1]. The overexpression of Group I *LEA* (*OsEm1*) in rice increased the ABA sensitivity of the transgenics and provided osmotic stress tolerance [12]. The role of *LEA14* has been linked with salt tolerance in *Arabidopsis thaliana* and *Setaria italica* [13,14].The role of *CaLEA1* has been reported in the sealing of stomata, and an increased expressions of drought and salt-responsive genes were observed in *CaLEA1* overexpression lines [15]. Recently, the role of Drought-Induced Late embryogenesis abundant protein 1 (*DIL1*) from *Capsicum annum* has been demonstrated to positively regulate ABA signaling and drought stress tolerance [16]. Interestingly, *LEA3* has been reported to function in lipid accumulation in *Brassica napus* and *Arabidopsis thaliana* by improving the photosynthetic efficiency of plants and reducing ROS levels [17].

Amongst all the LEA members, *Hordeum vulgare* aleurone *1* (*HVA1*), which belongs to group 3 LEA, is one of the most studied members. It has been extensively used in the transformation of numerous plants to improve their abiotic stress tolerance. The overexpression of *HVA1* in *Triticum aestivum* caused higher biomass production and water use efficiency under drought stress [18]. Similarly, Bahieldin et al. in 2005, reported an enhanced drought tolerance of transgenic wheat stably expressing the *HVA1* gene under field conditions [19]. When overexpressed in rice cv. Nippponbare, an increased tolerance to water deficit and salinity was observed; analysis of T2 generation further indicated that better cell membrane protection might contribute toward the tolerance against prolonged drought stress [20,21]. Its overexpression in rice cv. Pusa Basmati 1 also highlighted improved cell integrity as a factor contributing to salt and drought tolerance mechanisms [22]. Besides this, *HVA1* overexpression under the control of a stress-inducible promoter not only led to tolerance toward various abiotic stresses but also improved the root architecture [23]. The transformation of three oat cultivars with *HVA1* led to the osmotic stress tolerance [24]. Similarly, a constitutive or stress-inducible expression of *HVA1* in creeping bentgrass enhanced the turf quality and caused lower leaf wilting under water-deficient conditions [25]. Lal et al. in 2008 reported that *HVA1* constitutive overexpression in mulberry provided protection and stability of the plasma membrane and chloroplastic membranes under drought and salt stress [26]. Moreover, these plants were found to be tolerant to even cold stress conditions [27]. Enhanced drought tolerance and root length were reported in the case of transgenic *Phaseolus vulgaris* harboring the *HVA1* gene [28]. All these studies together highlight the potential role of *HVA1* in stress regulatory pathways. However, none of these studies have provided the molecular mechanism that lead to the stress tolerance. Thus, it is important to investigate the genes that function downstream to HVA1 in imparting resistance against multiple stresses.

Doubled haploid (DH) plants represent pure homozygous lines and are thus an important tool for biological studies and breeding. These plants are produced by culturing microspores and megaspores followed by chromosomal doubling [29]. The advantage of achieving complete homozygosity in one generation makes DH technology better than conventional breeding. This significantly reduces the time required for the production of pure lines. The complete homozygous nature of DH plants allows more precise phenotyping and helps in genetic mapping and gene function studies [29]. Wheat is one of the important cereal crops and thus a prime target for the improvement of stress tolerance. Previously, Chauhan and Khurana in 2011 demonstrated the use of anther culture for the production of DH plants overexpressing the *HVA1* gene [30]. These transgenic DH plants were found to be stable and showed a higher tolerance toward simulated water stress. Moreover, these DH lines represent true isogenic lines that are an ideal material to investigate the influence of a single gene on the functioning of the recipient plant. Therefore, for identifying the underlying molecular mechanisms involved in providing tolerance to these transgenics, they were further characterized. In the present study, the mechanism behind the drought tolerance of the DH plants (T7-T8 generation) was analyzed by using RNA-seq analysis. Apart from drought, the transgenic plants also showed heat stress tolerance at both vegetative and reproductive stages. The transcriptome analysis identified the upregulation of many heat-stress-responsive genes, which were validated by RT-PCR and were seen to contribute toward thermotolerance of the transgenic plants. Thus, this study indicates the potential role of *HVA1* in conferring heat stress tolerance together with drought stress in an important crop such as wheat.

## 2. Material and Methods

### 2.1. Phylogenetic Analysis

The multiple sequence alignment of the HVA1 protein along with the LEA3 protein sequences of *Triticum aestivum*, *Zea mays*, *Oryza sativa*, *Arabidopsis thaliana*, and *Solanum tuberosum* was performed using the MUSCLE program. The protein sequences were downloaded from the NCBI database and the neighbor-joining (NJ) phylogenetic tree was constructed by using MEGA 7 software. 

### 2.2. Plant Material and Stress Treatments

Bread wheat (*Triticum aestivum*) cultivar CPAN was used in this study. DH transgenic plants overexpressing *HVA1* were generated by an anther culture-based approach [30]. The protocol briefly consisted of a liquid culture phase for haploid embryo induction followed by *Agrobacterium*-mediated transformation and plantlet regeneration on gelled medium. The transgenic plants were confirmed and then multiplied for several generations [30]. Seeds of both T7- and T8-generation DH and wild type (WT) were surface-sterilized by using 4% sodium hypochlorite for 15 min followed by 4–5 washes with autoclaved water. For drought stress treatment, the seeds were germinated on a cotton bed in a growth chamber maintained at a 24/20 °C daily temperature under a daily cycle of 16 h light/8 h dark photoperiod having 200–300 μmol m^−2^ s^−1^ of light intensity. Ten-day-old seedlings were subjected to 200 mM mannitol for 24 h for drought stress and then allowed to recover [31,32]. The plants were photographed after 2 days of recovery. For the leaf-disc assay, the leaves of one-month-old transgenic and WT plants were cut into small pieces of equal sizes and were then floated onto 200 mM mannitol solution [33]. The segments were then incubated at a 24/20 °C daily temperature under a daily cycle of 16 h light/8 h dark photoperiod. The photographs of the leaf segments were taken after the 4 days of incubation. For giving drought stress at the vegetative and reproductive stage, potted plants (both transgenic and the WT) at Zadok stage Z30 and at the anthesis stage, i.e., Zadok stage 60–64, were selected and the plants were supplemented with 200 mM mannitol solution for 24 h [31]. After the drought stress, the leaf and the spike tissue were harvested and frozen in liquid nitrogen immediately for the RNA-seq analysis. Similarly, for heat stress treatments at vegetative and reproductive stages, plants at Zadok stage 30 and Zadok stage 60 were subjected to 42 °C for 2 h and 4 h, respectively [31,34]. The leaf and the spike tissue were harvested immediately after stress for the RNA-seq analysis. For further analysis at the reproductive stage, the plants were allowed to recover for 2 days after which photographs of the plants and leaves were recorded. Photographs were also recorded at the time of seed harvesting to observe the difference between the spikes and seeds of DH and WT plants. For expression profiling of *HVA1* in barley, ten-day-old seedlings were given drought stress by subjecting them to 200 mM mannitol solution for 1 h and 3 h [35,36]. For heat stress treatment, the seedlings were given 42 °C for 1 h and 3 h [37]. Seedlings were immediately frozen in liquid nitrogen after the stress treatments for RNA isolation.

For ABA treatment, seeds of both WT and transgenic DH were surface-sterilized by using 4% sodium hypochlorite for 15 min followed by 4–5 washes with autoclaved water. The seeds were allowed to dry inside the laminar air flow and were then plated onto media containing 5 µM ABA and 7 µM ABA [38,39]. The phenotype was observed after 4 and 7 days, respectively.

### 2.3. Histochemical ROS Detection and Quantification of H_2_O_2_

For analyzing the superoxide levels in the DH and the WT plants after heat stress, staining with Nitro Blue Tetrazolium (NBT) was performed according to Meena et al. (2020). For this, one-month-old plants of wheat were subjected to 42 °C for 2 h and then allowed to recover for 2 days after which the overnight staining of the small pieces of the leaves was performed with NBT (2 mM NBT powder, 20 mM phosphate buffer). The leaves were then washed with water and subjected to chlorophyll removal by dipping them in bleaching solution (ethanol, acetic acid, and glycerol in a ratio of 3:1:1). Similarly for hydrogen peroxide (H_2_O_2_) detection, DAB staining was performed according to [40]. The leaves after heat stress recovery were incubated in freshly prepared 3,3′-diaminobenzidine (DAB) solution (1 mg mL^−1^ DAB in Tris acetate buffer (pH 3.8)) in the dark for 18 h at 25 °C. The stained samples were then subjected to bleaching solution. The leaves were visualized under a bright field light microscope (Leica) and pictures were taken for comparison of ROS in the transgenic and WT plants. H_2_O_2_ was quantified according to [41]. For this, one hundred milligrams of the leaf tissue was ground using liquid nitrogen and was homogenized in 2 mL of 0.1% TCA. The homogenate was then centrifuged at 13,000× *g* for 20 min at 4°C. The supernatant was taken and mixed with an equal volume of 10 mM phosphate buffer (pH 7.0) and double volume of 1 M potassium iodide. The reaction was then incubated in the dark for 1 h at room temperature, after which its absorption was measured at 390 nm. The amount of H_2_O_2_ was calculated by using the standard curve. 

### 2.4. RNA Isolation and RNA-Seq Analysis

Total RNA was extracted using the RNeasy plant mini kit (Qiagen, Germany) according to the manufacturer’s protocol, which included DNaseI treatment and removal of genomic DNA contamination. Equal quantities of total RNA from three biological replicates for both treated and untreated DH plants were then pooled. Paired-end cDNA library preparation and sequencing was carried out by SciGenom, India. Briefly, the quality and quantity of the RNA samples were analyzed with an Agilent 2100 Bioanalyzer (Agilent Technologies, Santa Clara, CA, USA). Samples with RNA integrity values between 6.0 and 10.0 (RIN) were used for the RNA-seq analysis. After passing all the quality criteria, 1µg of the RNA per sample was used to purify polyA-tailed mRNA using polyT oligo-attached magnetic beads. The mRNA was fragmented (100–140 bp) and the first and second strand of cDNA synthesis was performed. The cDNA fragments were end-repaired followed by adaptor ligation for PCR purification and enrichment to create the final cDNA library. The library was then sequenced in the Hiseq 4000 platform instrument (Illumina, San Diego, CA, USA). 

The raw reads obtained after sequencing were filtered to remove adapter sequences and low-quality sequences by using Cutadapt. The RNA-seq reads were then mapped to the *Triticum aestivum* genome sequence obtained from Ensembl (IWGSC) using Trinity 2.0.6 software with default parameters [42]. The transcripts generated by Trinity were used for differential expression analysis. RSEM 1.2.7 software was utilized for the transcript abundance estimation and the FPKM (fragment per kilobase of exon fragments per million mapped) values were obtained. Differential expression was carried out by EdgeR 2.14 software with default parameters [43]. The expression pattern of the transcripts in each sample were restricted to transcripts with significant differential expressions (*p*-value 0.05, Fold change log2 scale).

### 2.5. Validation of Selected Genes by qRT-PCR 

Total RNA was extracted using the TRIzol Reagent (Ambion) according to the manufacturer’s protocol. An amount of 2 µg of the isolated RNA was converted into cDNA using the Applied Biosystems^TM^ High-Capacity cDNA Reverse transcription kit (Thermo Fisher Scientific, Lithuania). Quantitative Real-Time PCR (qRT-PCR) was conducted using SYBR Green (Applied Biosystems) in the QuantStudio^TM^ 3 Real-Time PCR system (Thermo Fisher Scientific) to study the expression of selected genes (primer sequences provided in Supplementary Table S1) with three biological and three technical replicates. *GAPDH* and *RNase L inhibitor like protein* (*RLI*) were used as the internal control for the analysis [44,45]. Relative gene expression was calculated according to the 2^−ΔΔCT^ method [46]. The real time data presented in graphs depict the mean ± standard deviation of mean (SD). The distribution of the data was assumed to be normal and, thus, the paired Student’s *t*-test was used for the statistical analyses of results. Statistical significant differences were shown at *p* ≤ 0.05 (marked *), *p* ≤ 0.01 (marked **), and *p* ≤ 0.001 (marked ***).

## 3. Results

### 3.1. Phylogenetic Analysis and Expression Profile of HVA1

**Figure 1:**
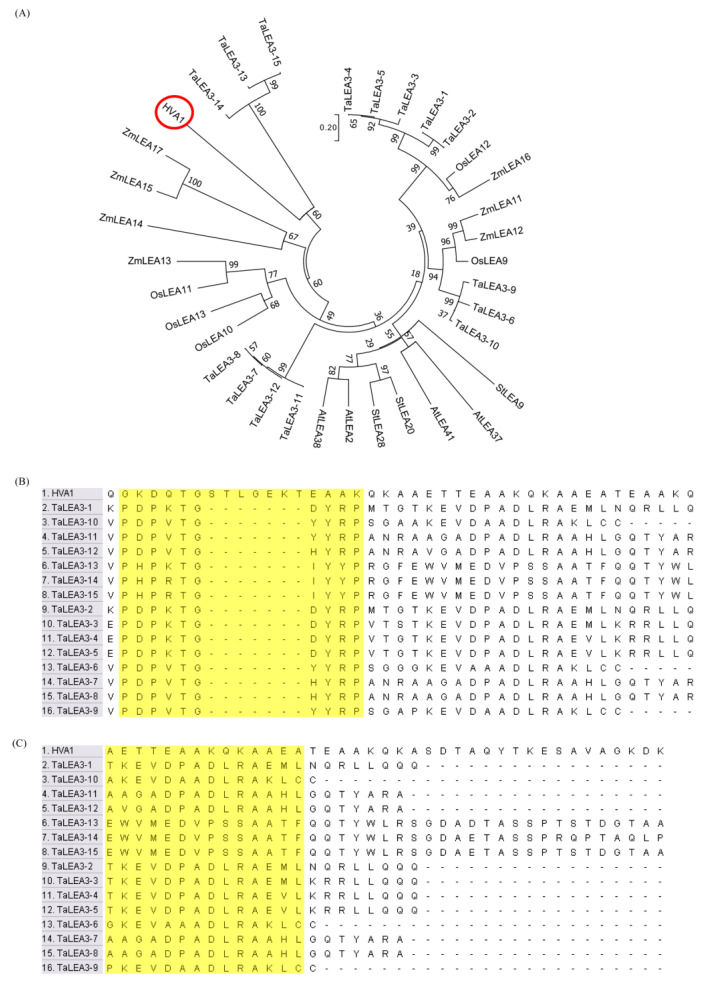
Phylogenetic analysis and sequence alignment of HVA1 and LEA3 proteins from other plant species. (**A**) Phylogenetic tree of HVA1 protein and LEA3 proteins from other plants. The unrooted tree was made using MEGA (version 7) with the neighbor joining method (1000 replicates). (**B**,**C**) Amino acid sequence alignment of W motif and EDVMP motif of HVA1 and TaLEA3 proteins.

In order to understand the molecular evolution of the *HVA1* gene, a phylogenetic tree was constructed of LEA3 protein sequences from different plant species such as *Triticum aestivum*, *Zea mays*, *Oryza sativa*, *Arabidopsis thaliana*, and *Solanum tuberosum* (Figure 1A). HVA1 was found to be closest to *Triticum aestivum* LEA3 proteins. Although 15 members of LEA3 were identified in wheat, HVA1 was observed to group with three members of TaLEA3, i.e., (TaLEA3-13, TaLEA3-14, and TaLEA3-15). Moreover, LEA3 proteins from various plant species are known to predominantly have five conserved motifs [47]. Sequence alignment showed that among the five conserved motifs, all the TaLEA3 proteins had the W-motif and the EDVMP motif (Figure 1B,C); however, the sequence of these two motifs was not fully conserved in the case of HVA1. This might have occurred due to the evolutionary differences between *Triticum aestivum* and *Hordeum vulgare.*

**Figure 2:**
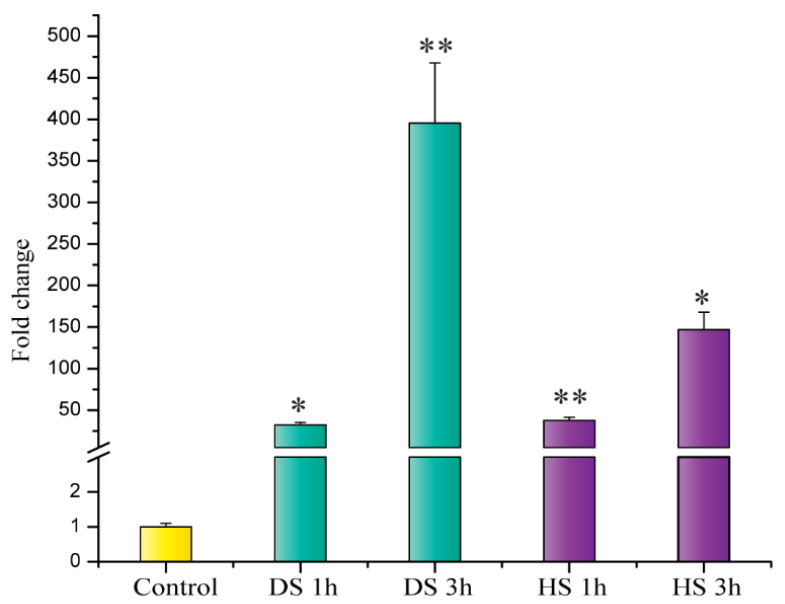
Expression analysis of *HVA1* under drought and heat stress conditions in *Hordeum vulgare* seedlings at different time points. Relative fold change was checked by qRT-PCR. Graph was plotted using three biological and three technical replicates. Error bars indicate values ± SD. Asterisks on the top of the error bars represent the significance value (Student’s *t*-test; *p* value ≤ 0.05 (marked *); *p* ≤ 0.01 (marked **)). (DS—Drought stress; HS—Heat stress).

To investigate the expression pattern of *HVA1* in barley under stress conditions, ten-day-old seedlings were subjected to drought and heat stress. As observed in Figure 2, *HVA1* was found to express significantly within 1 h of drought stress and it subsequently peaked at 3 h. Interestingly, in the case of heat stress as well, levels of *HVA1* were found to be significantly increased within 1 h and 3 h of the treatment. This indicated that *HVA1* is inducible by both drought and heat stress.

### 3.2. Phenotypic Analysis of HVA1 DH Plants Showed Improved Drought Tolerance 

**Figure 3:**
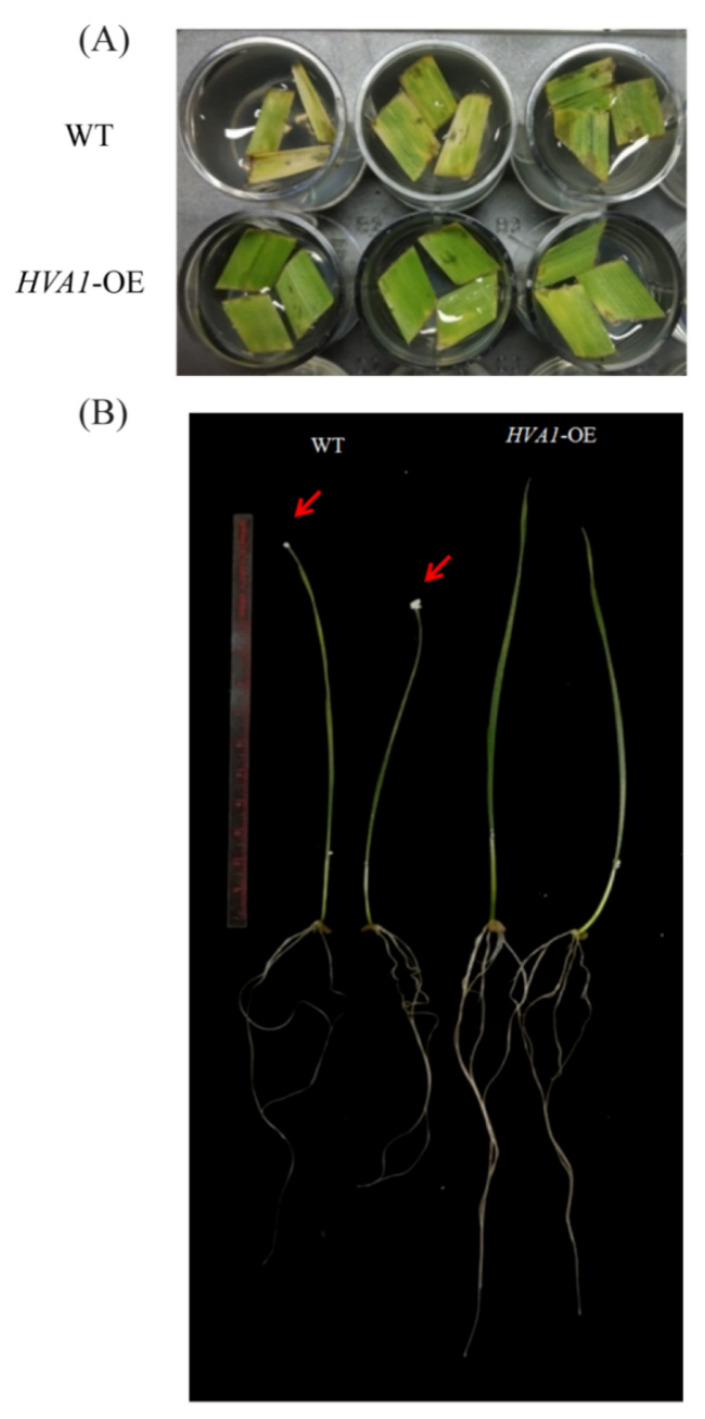
Phenotype of *HVA1*-overexpression transgenic wheat DH plants under drought stress. (**A**) Leaf senescence assay. Leaf segments of WT and transgenic plants were incubated in 200 mM mannitol. (**B**) Ten-day-old seedlings of WT and transgenic DH plants were supplemented with 200 mM mannitol for 24 h and then allowed to recover for 2 days.

In wheat, *HVA1* DH plants at T4 generation have been reported to show tolerance against stimulated water stress [30]. To further assess the drought tolerance of these plants, a leaf senescence assay was performed at T7 and T8 generation. As observed in Figure 3A, faster senescence was observed in WT as compared to the *HVA1* overexpression lines. Apart from this, the ten-day-old seedlings were given drought stress with 200 mM mannitol. In the case of WT, the leaf showed senescence, root growth was inhibited, and it had salt deposited on the leaf tips. On the other hand, the overexpression lines performed better as evident by its greener leaf, robust root growth, and almost no salt deposition on the leaf tips (Figure 3B). Thus, the wheat DH plants showed significant drought tolerance.

### 3.3. Transcriptome Analysis of DH Plants after Drought Stress 

**Figure 4:**
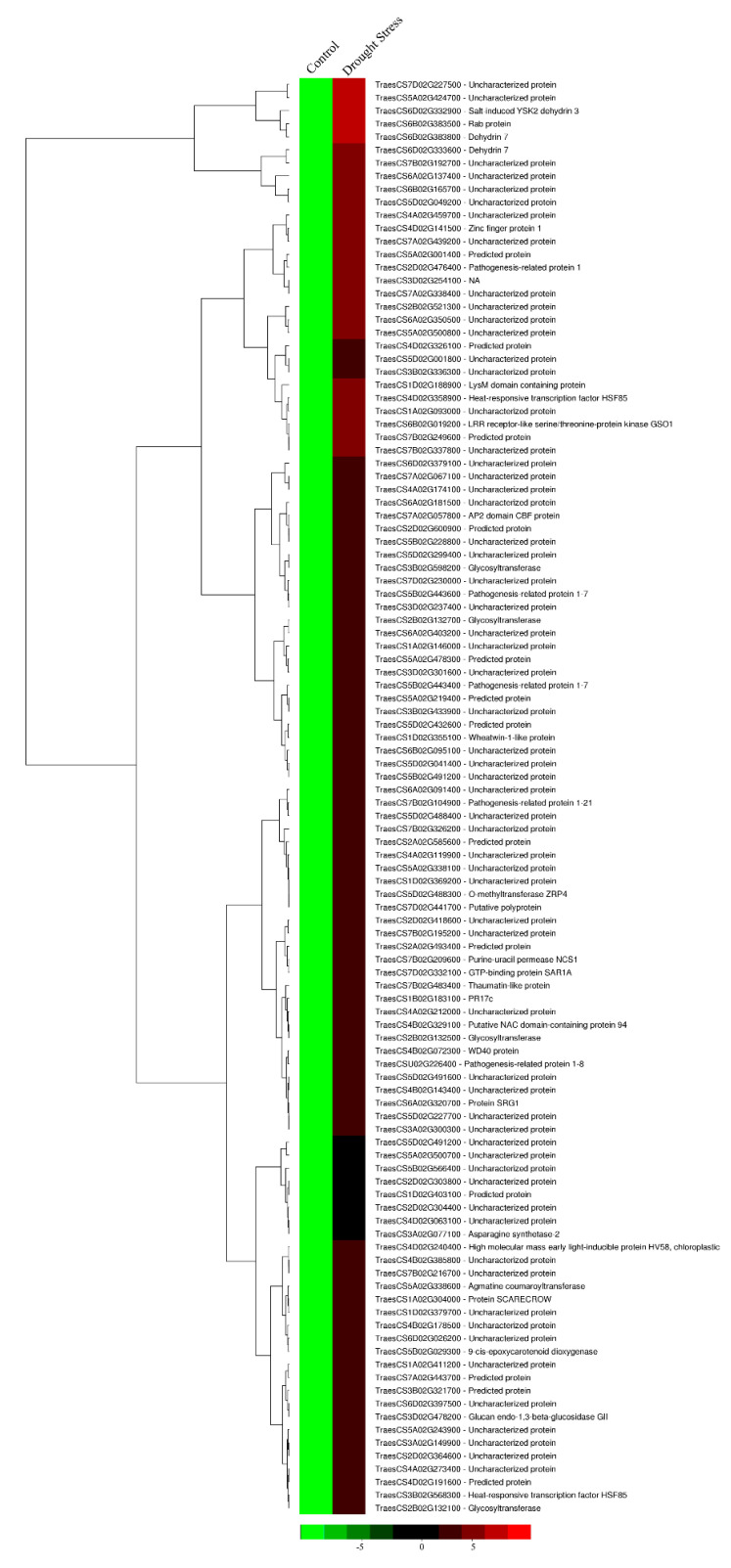
Heatmap displaying the differentially expressed genes upregulated in *HVA1*-overexpression wheat DH transgenics under drought stress conditions (200 mM mannitol) at the vegetative stage based on the FPKM data. Column corresponds to the experimental design and row corresponds to the gene. The color key represents FPKM-normalized log10-transformed counts.

To understand the drought tolerance mechanism in the *HVA1* overexpression lines, transcriptome analysis was performed both at the vegetative stage and at the reproductive stages in the drought-stressed plants. Comparison between non-stressed and stressed DH plants at the vegetative stage showed a total of 426 Differentially Expressed Genes (DEGs). The upregulated genes encoded for various types of proteins such as dehydrins (TraesCS6D02G332900; TraesCS6B02G383800), Rab (TraesCS6B02G383500), AP2 domain containing CBF protein (known as DREB) (TraesCS7A02G057800), Glycosyltransferase (TraesCS2B02G132700), O-methyltransferase (TraesCS5D02G488300), pathogenesis-related protein (TraesCS2D02G476400), Zinc finger protein 1 (TraesCS4D02G141500), NAC domain-containing protein (TraesCS4B02G212000), heat-responsive transcription factor 85 (TraesCS4D02G358900), and LRR receptor-like serine/threonine-protein kinase (TaresCS6B02G019200) (Figure 4). In contrast, the downregulated genes list included genes such as *Auxin-responsive protein SAUR36* (TraesCS7D02G273500), various genes coding for histones such as *Histone H2B* (TraesCS6D02G040200), *Histone H4* (TraesCS1B02G380200) and *Histone H2A* (TraesCS6B02G058400), and enzymes such as *Phosphatase* (TraesCS4A02G400600), *Arginine decarboxylase* (TraesCS1D02G012300), *Esterase/lipase* (TraesCS6D02G372000), *Pectin acetylesterase* (TraesCS3D02G539100), *MYB75* (TraesCS1D02G084400), and *Non-specific lipid transfer protein* (TraesCS3D02G338300) (Figure S1).

**Figure 5:**
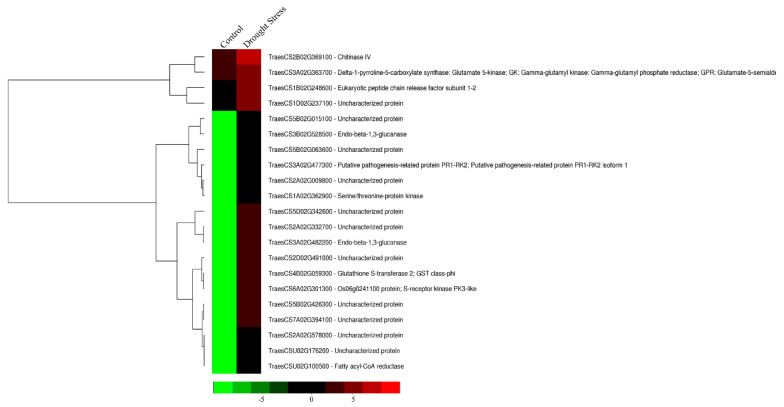
Heatmap displaying the differentially expressed genes upregulated in *HVA1*-overexpression wheat DH transgenics under drought stress conditions (200 mM mannitol) at the reproductive stage based on the FPKM data. Column corresponds to the experimental design and row corresponds to the gene. The color key represents FPKM-normalized log10-transformed counts.

At the reproductive stage, the upregulated genes included *Chintinase IV* (TraesCS2B02G369100), *Fatty acyl-CoA reductase* (TraesCSU02G10500), *Serine-threonine protein kinase* (TraesCS1A02G362900), *Glutathione-S-transferase* (TraesCS4B02G059300), and *Endo-beta-1,3-glucanase* (TraesCS3B02G528500) (Figure 5). The downregulated genes included *Flavin-containing monooxygenase* (TraesCS3A02G149500) and *Endo-1,4- beta-Xylanase C* (TraesCS2A02G584800) (Figure S2).

**Figure 6:**
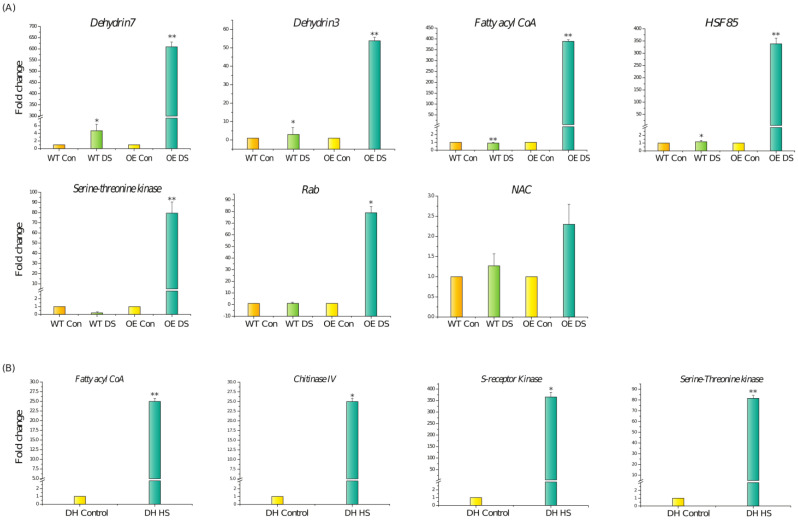
(**A**) Validation and expression analysis of genes in WT and wheat transgenic DH plants under control and drought stress conditions at the vegetative stage. Expression was checked in the leaf tissue using qRT-PCR. (**B**)Validation and expression analysis of genes in wheat transgenic DH plants under control and drought stress conditions at the reproductive stage. Expression was checked in the spike tissue using qRT-PCR. Relative fold change was calculated. Graphs were plotted using three biological and three technical replicates. Error bars indicate values ± SD. Asterisks on the top of the error bars represent the significance value (Student’s *t*-test; *p* value ≤ 0.05 (marked *); *p* ≤ 0.01 (marked **)).

Amongst the upregulated genes, some of them were validated by quantitative real-time PCR. At the vegetative stage, a significant upregulation of the *Dehydrin 3*, *Dehydrin 7*, and *Rab* genes was observed under drought stress in DH plants. However, their expression did not increase much in the WT plants under drought stress. Transcript levels of *HSF85*, *Fattyacyl CoA*, *NAC*, and *Serine-threonine protein kinase* were also found to be higher in the case of transgenic as compared to WT under drought stress conditions (Figure 6A). At the reproductive stage, the expression of genes such as *Chitinase IV*, *Fattyacyl CoA*, *S-receptor kinase*, and *Ser-threonine kinase* were found to be significantly upregulated in the DH plants in comparison to the DH control plants, thereby validating the results of RNA-seq data (Figure 6B).

### 3.4. HVA1 Overexpression Promoted Thermotolerance in Wheat

**Figure 7:**
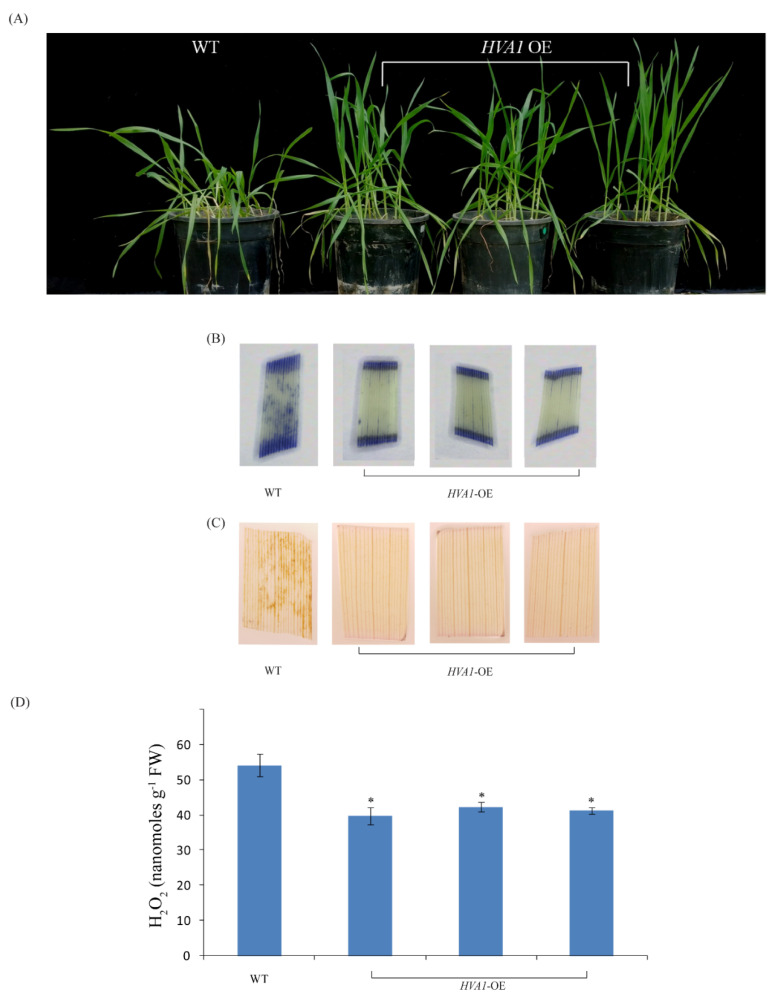
Phenotype of *HVA1*-overexpression wheat transgenic DH plants under heat stress at vegetative stage. (**A**) Photograph of the one-month-old WT and transgenic DH plants after 2 days of recovery following heat stress. (**B**) Detection of superoxide ions in WT and transgenic DH plants by NBT staining. (**C**) Detection of hydrogen peroxide in WT and transgenic DH plants by DAB staining. The presence of blue formazan precipitate and dark brown DAB precipitate indicates higher superoxide and hydrogen peroxide accumulation, respectively. (**D**) Quantification of the hydrogen peroxide levels in the WT and DH plants. The data represent mean ± SE of three biological replicates. Asterisks on the top of the error bars represent the significance value (Student’s *t*-test; *p* value ≤ 0.05 (marked *)).

A recent genome-wide study by [48] identified *TaLEA3* members to be upregulated by heat stress and provide thermotolerance when overexpressed in *E. coli* and yeast. Taking hint from this, we checked the thermotolerance of the DH plants. At the vegetative stage, when exposed to 42 °C, the DH plants recovered better in comparison to the WT plants (Figure 7A). To assess the recovery of plants, the level of ROS (superoxide anions and hydrogen peroxide) was checked in both WT and DH plants by NBT and DAB staining. Lower ROS levels were observed in the DH leaves as compared to the WT (Figure 7B,C). Moreover, the level of hydrogen peroxide was also quantified in the transgenic and WT plants (Figure 7D). The DH plants showed a lower hydrogen peroxide accumulation after heat stress as compared to the WT.

**Figure 8:**
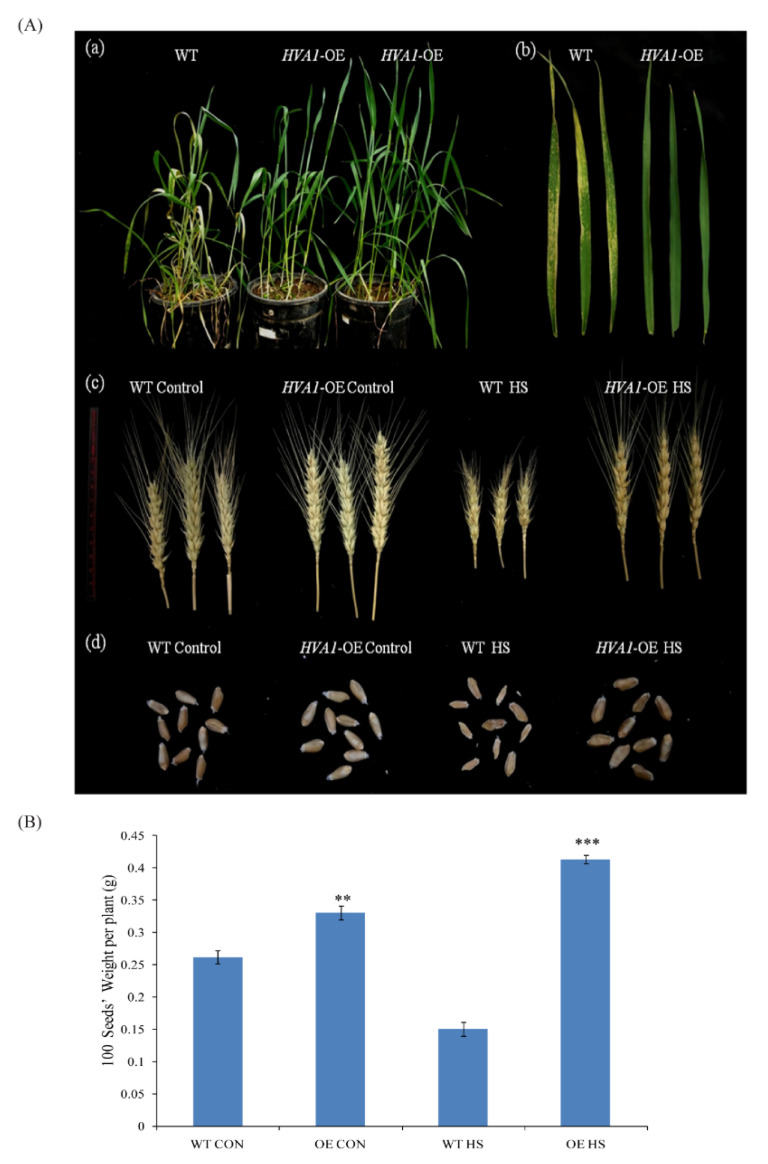
(**A**)Phenotype of *HVA1*-overexpression wheat transgenic DH plants under heat stress at reproductive stage. (**a**) Photograph of the WT and transgenic DH plants (heading stage) after 2 days of recovery following heat stress. (**b**) Photograph of the WT and transgenic DH leaves showing the oxidative damage after recovery. (**c**) Photograph of the WT and transgenic DH spikes. (**d**) Photograph of the WT and transgenic DH spikes and seeds under control and heat stress conditions. (**B**) Graph representing seed weight of WT and transgenic DH plants under control and heat stress conditions. The data represent the mean ± SE of three biological replicates. Asterisks on the top of the error bars represent the significance value (Students *t*-test; *p* ≤ 0.01 (marked **); *p* ≤ 0.001 (marked ***)).

At the reproductive stage when the plants were subjected to heat stress conditions, a better performance of DH plants was also observed, as depicted in Figure 8. The leaves of the WT showed more oxidative damage as compared to the DH plants (Figure 8A(b)). Moreover, the transgenic plants had larger spikes as compared to the heat-stressed WT plants (Figure 8A(c)). Transgenic DH plants were found to have a larger seed size and more seed weight in comparison to WT (Figure 8A(d),B).

### 3.5. Transcriptome Analysis of DH Plants after Heat Stress

**Figure 9:**
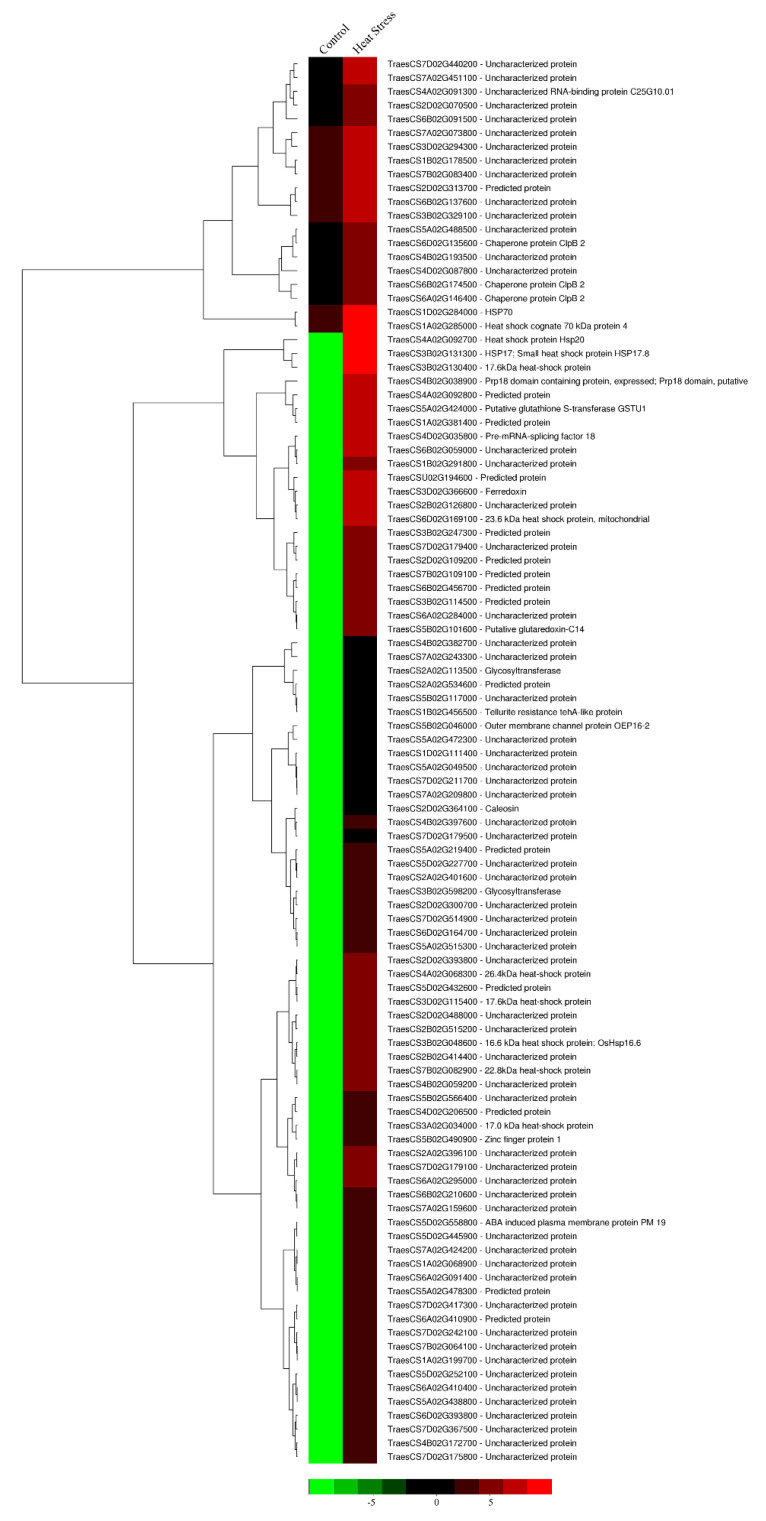
Heatmap displaying the differentially expressed genes upregulated in *HVA1*-overexpression wheat DH transgenics under heat stress conditions at the vegetative stage based on the FPKM data. Column corresponds to the experimental design and row corresponds to the gene. The color key represents FPKM-normalized log10-transformed counts.

To investigate the molecular mechanisms contributing toward the heat stress tolerance, RNA-seq analysis of the transgenic DH plants after heat stress treatment was undertaken and was compared with the transgenic DH control plants. A total of 51 genes were found to be differentially expressed under control and heat stress conditions at the vegetative stage. The upregulated genes included various HSPs such as *HSP70* (TraesCS1D02G284000), *HSP17* (TraesCS3B02G131300), *HSP20* (TraesCS4A02G092700), *ClpB 2* (TraesCS6A02G146400), *Glutathione -S-transferase* (TraesCS5A02G424000), *Ferrodoxin* (TraesCS3D02G366600), *ABA-induced plasma membrane protein PM19* (TraesCS5D02G558800), *Caleosin* (TraesCS2D02G364100), *Cytochrome P450* (TraesCS2A02G534600), and *Haem peroxidase* (TraesCS2D02G070500) (Figure 9). The downregulated genes were found to be uncharacterized as they had no special domain to identify their function (Figure S3A).

**Figure 10:**
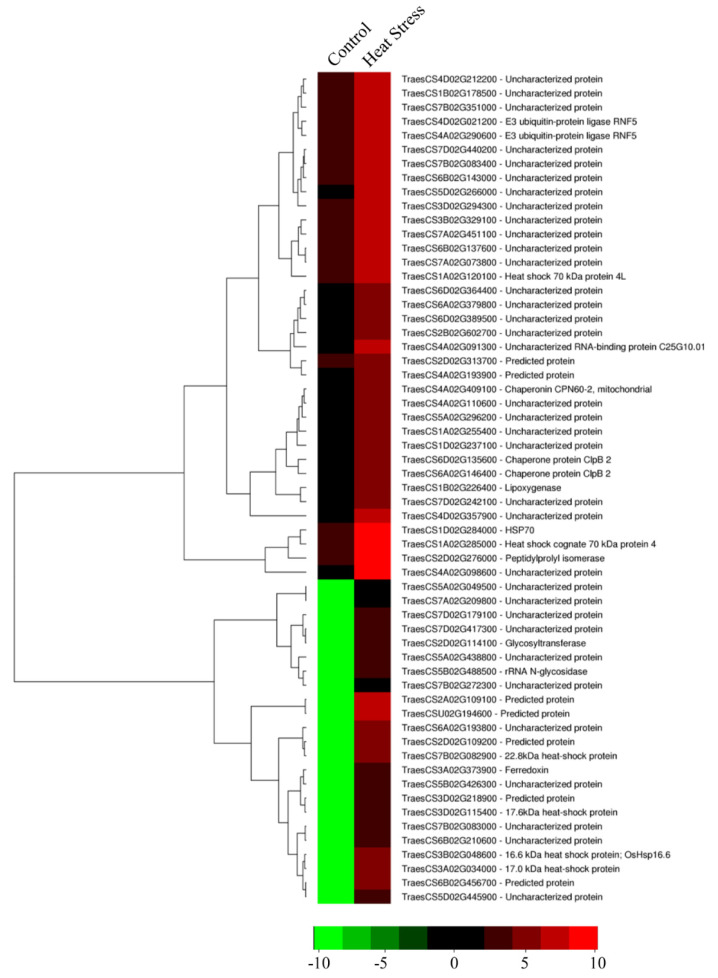
Heatmap displaying the differentially expressed genes upregulated in *HVA1*-overexpression wheat DH transgenics under heat stress conditions at the reproductive stage based on the FPKM data. Column corresponds to the experimental design and row corresponds to the gene. The color key represents FPKM-normalized log10-transformed counts.

At the reproductive stage, the upregulated genes included *Lipoxygenase* (TraesCS1B02G226400), *Chaperonin CPN60-2* (TraesCS4A02G409100), *Mitochondrial carrier protein* (TraesCS7A02G209800), *E3 ubiquitin ligase RNF5* (TraesCS4D02G021200), and *Peptidylprolyl isomerase* (TraesCS2D02G276000) (Figure 10). The downregulated genes mainly had uncharacterized proteins (Figure S3B).

**Figure 11:**
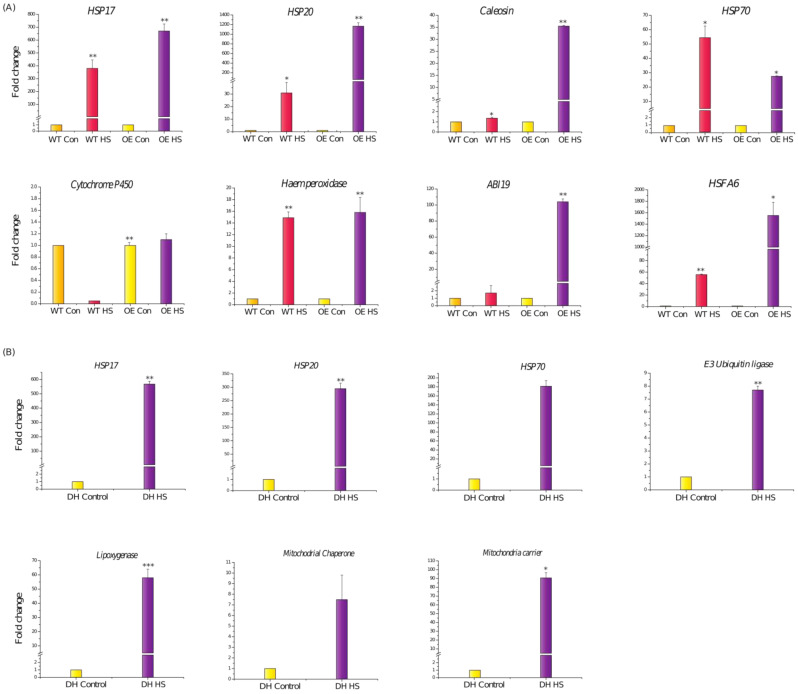
(**A**) Validation and expression analysis of genes in WT and wheat transgenic DH plants under control and heat stress conditions at the vegetative stage. Expression was checked in the leaf tissue using qRT-PCR. (**B**) Validation and expression analysis of genes in transgenic wheat DH plants under control and heat stress conditions at the reproductive stage. Expression was checked in the spike tissue using qRT-PCR. Relative fold change was calculated. Graphs were plotted using three biological and three technical replicates. Error bars indicate values ± SD. Asterisks on the top of the error bars represent the significance value (Student’s *t*-test; *p* value ≤ 0.05 (marked *); *p* ≤ 0.01 (marked **); *p* ≤ 0.001 (marked ***)).

Some of the genes identified in the transcriptome data were further confirmed by qRT-PCR. At the vegetative stage, a significant upregulation of the *TaHSP17*, *TaHSP20*, and *TaHsfA6* genes under heat stress was observed in the DH transgenic plants in comparison to their expression in WT plants (Figure 11A). Moreover, higher expression levels of *Caleosin*, *Haem peroxidase*, and *ABA-induced plasma membrane protein PM 19 (ABI19)* were also observed in the heat-stressed DH in comparison to their almost negligible expression in the heat-stressed WT plants (Figure 11A). Even at reproductive stage, the expression profiles of various HSPs such as *TaHSP17*, *TaHSP20*, and *TaHSP70* were found to upregulated in the spike tissue of DH after the heat stress treatment as compared to the control (Figure 11B). Apart from HSPs, higher transcript levels of *Mitochondrial carrier*, *Lipoxygenase*, *E3-Ubiquitin ligase RNF5*, and *Mitochondrial chaperonin CPN60-2* were also found (Figure 11B).

### 3.6. Enhanced Sensitivity of HVA1-Overexpression Plants to ABA

**Figure 12:**
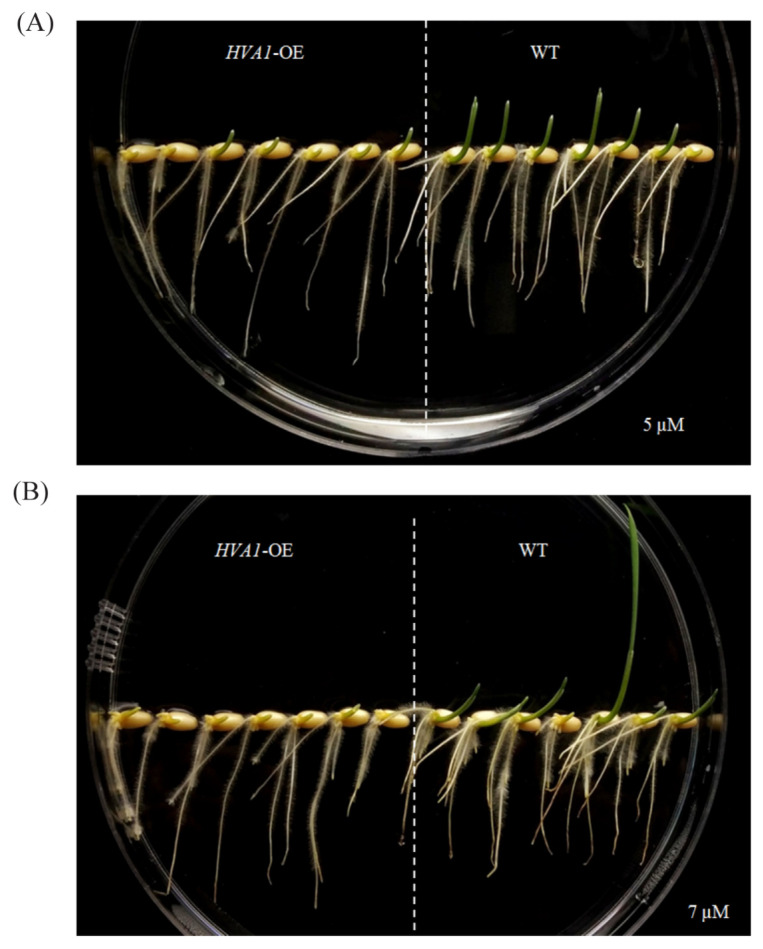
Phenotype of *HVA1*-overexpression wheat transgenic DH plants on ABA containing medium. (**A**) Seeds of WT and transgenic DH were germinated on medium containing 5 µM of ABA. Phenotype was observed after 4 days. (**B**) Seeds of WT and transgenic DH were germinated on medium containing 7 µM of ABA. Phenotype was observed after 7 days.

ABA is one of the key hormones known to accumulate during stress conditions, particularly drought stress [49]. As the DH plants showed the stress-tolerant phenotype, they were screened on ABA containing medium. The seeds of both WT and overexpression lines were germinated on 5 µM and 7 µM of ABA. As observed in Figure 12, the DH seeds were found to be ABA-sensitive on both concentrations of ABA as compared to the WT seeds that showed germination and growth after 4 and 7 days respectively. This suggests that *HVA1* positively modulates the drought and heat stress response via the ABA-mediated signaling pathway.

## 4. Discussion

LEA comprises one of the largest protein families that is widely diverse across the plant kingdom [50]. HVA1 is one of the first characterized and the most well studied LEA3 proteins from *Hordeum vulgare* [51]. Previous work by Chauhan and Khurana (2011) overexpressed *HVA1* in wheat by the use of DH technology, and the transgenic plants were found to be drought-tolerant [30]. However, the molecular mechanisms involved in providing tolerance to the plants remained unknown. DH technology provides the fastest and most efficient way of generating complete homozygous lines as compared to conventional inbred lines, which are known have residual heterozygosity. Complete homozygosity is not only important for breeding and studying gene functions, but it also offers a higher phenotype to genotype correlation [52]. Therefore, in this study, the DH plants that can be considered as true isogenic lines were analyzed and found to be both drought- and heat-tolerant. Moreover, as the genetic variation for heat and drought stress tolerance in elite wheat germplasm is limited, it may not provide sufficient resistance to develop cultivars through traditional breeding. Thus, genetic engineering provides a way to enhance both heat and drought stress resistance in wheat. Although there exist many differences between the wheat and barley genomes such as ploidy levels and other genetic differences, the heterologous overexpression of the *HVA1* gene provided stress tolerance in wheat. Therefore, it can be speculated that the overexpression of *TaLEA3* in wheat itself would also provide stress tolerance in a more effective way (due to the same genetic background). Thus, this study highlights *LEA3* as a potential gene for incorporating the simultaneous heat and drought tolerance trait in wheat.

Drought stress is known to accelerate leaf senescence, leading to a loss in photosynthesis and reduction in yield [53,54]. The drought stress tolerance of the DH plants was analyzed by a leaf disc senescence assay, which clearly showed that the leaves of the transgenic plants showed delayed senescence as compared to the WT plants (Figure 3A). Moreover, at the seedling stage, the DH plants showed the tolerant phenotype as compared to the WT when subjected to simulated drought stress (Figure 3B). These results suggested that the overexpression of *HVA1* contributes toward drought tolerance. This was found to be consistent with earlier reports wherein the overexpression of *LEA3* provided drought tolerance to various plants (such as creeping bentgrass, mulberry, *Arabidopsis thaliana*, and *Brassica napus*) and freezing tolerance in the case of yeast [17,25,27,55]. Thus, it could be concluded that *LEA3* has a conserved functional role in providing tolerance toward drought stress. To gain insight into its mechanism of action, transcriptome data were analyzed after drought stress treatment. Interestingly, the upregulation of transcription factors such as *NAC* and *DREB* was observed in the DH plants, whereas their expression was found to be low in the WT plants after the drought stress treatment (Figure 6A). This could be supported by the evidence in *Oryza sativa* and *Arabidopsis*, wherein, the *NAC* transcription factor by activating *DREB2A* contributes toward drought tolerance of the plants [56,57]. Interestingly, the levels of both *NAC* and *AP2 CBF/DREB* were found to be upregulated more in the transgenic DH plants as compared to the WT plants after drought stress. Therefore, it could be speculated that *HVA1* by interacting with other transcription factors either regulates the expression of both *NAC* and *DREB* independently, or it only regulates *NAC*, which then activates *DREB* to contribute toward the drought tolerance. Apart from transcription factors, expressions of *Rab* and *Dehydrin* genes were also found to be high in the DH plants. Rab and Dehydrin proteins have been reported to be involved in the drought signaling pathway and in protective functions against ROS generated during drought stress, respectively [58,59]. Therefore, higher expressions of *Rab* and *Dehydrin* genes might also provide drought tolerance to the DH plants. However, how *HVA1* is able to regulate these genes under drought stress conditions has become an area of future research.

Apart from drought stress, there are limited reports that have highlighted the role of *LEA* in the heat stress response. Therefore, the heat tolerance of DH plants was also analyzed at both the vegetative and reproductive stage. In comparison to WT plants, the DH plants recovered better after the heat stress (Figure 7A). ROS accumulation is known to occur after heat stress, which often leads to cellular damage [60,61]. The DH plants had a lower oxidative load as compared to the WT (Figure 7D). This observation could further be corroborated with the upregulation of *Haem peroxidase* in the transgenic plants as compared to the WT (Figure 11A). This suggest that the higher transcript levels of this antioxidant enzyme leads to accumulation of the enzyme which help the *HVA1* overexpression lines to survive better under heat stress by maintaining lower ROS levels. Consistent with this, *Ascorbate peroxidase* (*APX*), which belongs to class I *Haem-peroxidase*, has been shown to be heat-responsive in different plants, and its absence in *Arabidopsis* leads to sensitivity to heat stress [62,63,64]. Apart from this, group 3 LEA proteins are known to possess the 11-mer amino acid motif with the consensus sequence of TAQAAKEKAGE. Interestingly, this motif occurs nine times in the HVA1 protein, leading to alpha helical dimer formation, which is suitable to accommodate both positively and negatively charged ions [26].The ZmLEA3 protein has been found to bind metal ions, and the authors speculated that the binding of ions by ZmLEA3 contributes toward reducing oxidative damage and ion toxicity during abiotic stress [65]. Therefore, it is probable that HVA1 might reduce oxidative load inside the cell by binding to the metal ions through the alpha helical dimer.

Transgenic DH plants were found to be tolerant to heat stress even at the reproductive stage, as they had better spikes and higher seed weight in comparison to the WT (Figure 8A(c),B). This result was further supported by the transcriptome data wherein the upregulation of various HSPs was observed in the transgenic plants after heat stress (Figure 10). Moreover, the levels of expression of *HSP17* and *HSP20* were found to be higher in the DH plants (Figure 10 and Figure 11B). The role of small HSPs such as *TaHSP26* has been earlier documented to provide heat stress tolerance to the overexpression plants [66,67]. Therefore, significant upregulation of the small HSPs plays a major role in providing thermotolerance to the *HVA1-*overexpression plants. Apart from stress, it is well known that HSPs accumulate during seed maturation [68]. As seed formation was observed to be better in the case of DH plants after heat stress, it is possible that the higher expression of these HSPs in the seeds might have caused enhanced seed development under heat stress conditions. Further, higher expression levels of mitochondrial carriers and mitochondrial chaperone proteins in *HVA1*-overexpression plants highlight their function in the heat stress response to protect the mitochondrial components from degradation and proteolysis [69,70].

Interestingly, *ABI19*, one of the ABA-responsive genes, was found to be upregulated in the DH plants after heat stress (Figure 9). Its expression levels were found to be higher in the transgenic plants as compared to the WT (Figure 11A). A report by Huang and co-workers highlighted the role of *HsfA6b* connecting ABA signaling and the ABA-mediated heat stress response [71]. Therefore, the expression of *TaHsfA6* was also analyzed in the transgenic and WT plants under heat stress conditions. Interestingly, more transcript levels of *TaHsfA6* were observed in the case of DH plants (Figure 11A). Therefore, it could be speculated that *TaHsfA6* leads to the ABA-mediated heat stress response in the transgenic plants. This could be further supported by the ABA-sensitive phenotype of the transgenic plants (Figure 12). Thus, these results suggest that *HVA1* via the ABA-mediated pathway plays a role in heat stress.

**Figure 13:**
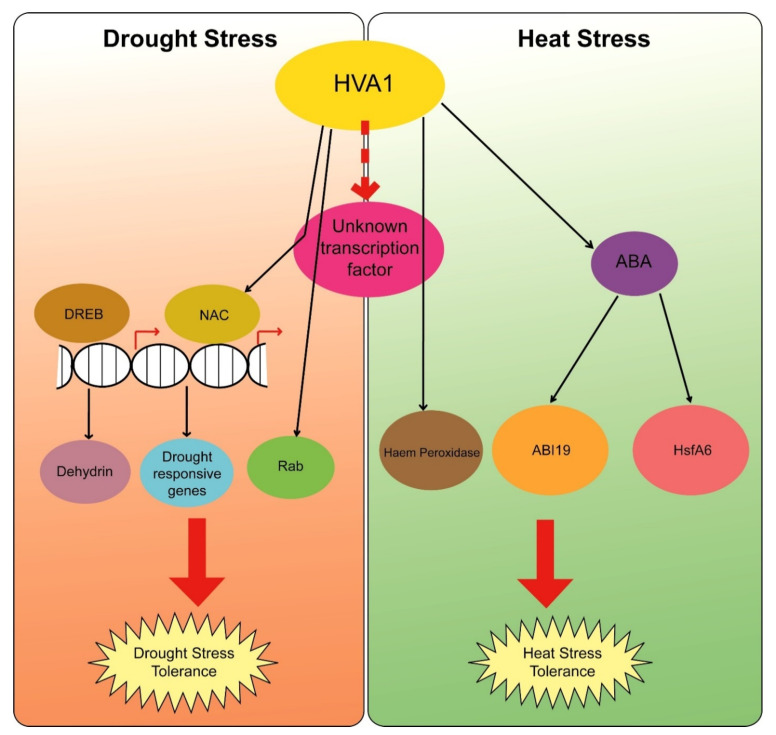
Hypothetical model for *HVA1* response under drought and heat stress. Under drought stress conditions, HVA1, by interacting with another unknown transcription factor, leads to the transcription of genes such as *NAC*, *DREB*, and *Rab*. These transcription factors further activate *Dehydrin* and other drought-responsive genes, which help in acquiring drought stress tolerance in plants. In the case of heat stress conditions, *HVA1* activates *HsfA6* and *ABI19* via the ABA-mediated pathway. HsfA6 then leads to the expression of HSPs, which play a major role in providing protection against heat stress.

In conclusion, *HVA1*, which belongs to the *LEA3* group, was found to play a major role in response to both drought and heat stress. However, as *HVA1* lacks DNA binding domain, it is suspected that it might interact with some transcription factor to activate master regulators such as *NAC*, *DREB*, and *HsfA6* to bring about both drought and heat stress tolerance. A hypothetical model summarizing the probable role of *HVA1* under both drought and heat stress conditions is presented in Figure 13. Upon drought stress, *HVA1* leads to the activation of genes such as *NAC*, *DREB*, and *Rab*. *NAC* and *DREB*, in turn, lead to the transcription of other drought-responsive genes such as *dehydrins,* which help in providing drought tolerance to plants. Similarly, under heat stress conditions, *HVA1* via the ABA-mediated pathway activates the expression of *TaHsfA6,* which leads to the accumulation of small Hsps, thereby attributing heat stress tolerance. Apart from this, *HVA1* also activates *ABI19* and *Haem peroxidase*, which also contributes toward the abiotic stress tolerance mechanism. However, the identification of *HVA1*-interacting partners (transcription factors) needs to be investigated further. Moreover, the mechanism of regulation of *HVA1* itself needs to be explored to understand how it works under both stresses. Overall, *HVA1* appears to play a role in providing multiple stress tolerance to the plant both at the vegetative and reproductive stages, by modulating the transcriptome at multiple levels.

## Data Availability

The RNA-seq data reported in this article have been submitted in the NCBI database (Bioproject PRJNA811517).

## Supplementary Materials

The following supporting information can be downloaded at https://www.mdpi.com/article/10.3390/cells11050912/s1, Figure S1: Heatmap displaying the differentially expressed genes down-regulated in *HVA1* overexpression wheat DH transgenics under drought stress conditions (200 mM mannitol) at the vegetative stage based on the FPKM data; Figure S2: Heatmap displaying the differentially expressed genes down-regulated in *HVA1* overexpression wheat DH transgenics under drought stress conditions (200 mM mannitol) at the reproductive stage based on the FPKM data; Figure S3: (A) Heatmap displaying the differentially expressed genes down-regulated in *HVA1* overexpression wheat DH transgenics under heat stress conditions at the vegetative stage based on the FPKM data. (B) Heatmap displaying the differentially expressed genes down-regulated in *HVA1* overexpression wheat DH transgenics under heat stress conditions at the reproductive stage based on the FPKM data; Table S1: List of primers used for the qPCR analysis.

## Abbreviations

ABA	abscisic acid
DH	doubled haploid
HVA1	Hordeum vulgare aleurone 1
LEA	late embryogenesis abundant
ROS	reactive oxygen species
WT	wildtype
