# Supplementary material for: Overexpression of HVA1 Enhances Drought and Heat Stress Tolerance in Triticum aestivum Doubled Haploid Plants
Source: Cells. 2022 Mar 7;11(5):912. doi: 10.3390/cells11050912 (PMC8909738; doi:10.3390/cells11050912)
Supplement: Supplementary file 1 [file cells-11-00912-s001.zip › cells-1629540-supplementary/Table S1.pdf]

Supplementary Table S1. List of primers used for the qPCR analysis

S.No	Primer Name	Primer Sequence
1	*TaHsfA6 RT F*	TAGACGTGGAGGTGGAGGAG
2	*TaHsfA6 RT R*	ATCTGAACTTCCGGAGCGTA
3	*TaABI19 RT F*	GTGTAAGCCAAGCCGGTGAT
4	*TaABI19 RT F*	CACACCGTACGTGACCCATACT
5	*TaHSP70 RT F*	TCGCCTACGGCCTTGACA
6	*TaHSP70 RT R*	ATGGTCAGCAGCGACACATC
7	*TaHSP17 RT F*	CGAGAATGCTGACATGGAGA
8	*TaHSP17 RT R*	ACCTGGACCTGGATGGTCTT
9	*Caleosin RT F*	TGTACGACGGCAGCCTCTTC
10	*Caleosin RT R*	GCTCCGACCATGCGAGTTT
11	*Cytochrome P-450 RT F*	AAAGTCGCAAACACCTCACC
12	*Cytochrome P-450 RT F*	GTGACGAGGAAGGAGTGGAG
13	*Haeme Peroxidase RT F*	GGGATTCTTCAGTTGCCATTG
14	*Haeme Peroxidase RT R*	TTGTCGGTCAAGCCCATTC
15	*AP2 domain CBF protein RT F*	TGTTCTTGAAAATTGGCGCTAA
16	*AP2 domain CBF protein RT R*	TCTGTAACCGCTCAGCAAACTG
17	*TaHSP20 RT F*	CATCGACTGGAAGGAGATGC
18	*TaHSP20 RT F*	CTTCTGCTCCTGCTCCTTGT
19	*Lipoxygenase RT F*	TGCGGATTTCTTTGTTGGTTT
20	*Lipoxygenase RT R*	CGTTTTGTGAGAGCTGTTTCCA
21	*Chaperonin CPN60-2, mitochondrial RT F*	GCCTACAGCAAACTTTGACCAA
22	*Chaperonin CPN60-2, mitochondrial RT R*	CCCCTTCTACACCCGCATT
23	*E3 ubiquitin-protein ligase RNF5 RT F*	TCTGGATGGTCTTCCTCTGCAT
24	*E3 ubiquitin-protein ligase RNF5 RT R*	GGGATGACCTCCCAACAATTG
25	*Ferrodoxin RT F*	GCACAAGGTGAAGCTCGTC
26	*Ferrodoxin RT R*	GCACGAGTAGGGCAGCTC
27	*Salt-induced YSK2 dehydrin 3 RT F*	CGGTCGCCACTTCTGAGAGT
28	*Salt-induced YSK2 dehydrin 3 RT R*	CACGCAAGTGAGCTAGGTGAAC
29	*Dehydrin 7 RT F*	CCAGGACACACCGGAATGAC
30	*Dehydrin 7 RT R*	CGCCAGCCAGGCTCAGT
31	*Rab protein RT F*	GGCCGCCACTTCTGAGAGT
32	*Rab protein RT F*	GGCTCACGTTGTATGGTGGAA
33	*HSF85 RT F*	TGGCTTTTTAGACGACGTGGTA
34	*HSF85 RT R*	TGGGCTGCTTCACCAAAAAT
35	*Putative NAC domain containing protein 94 RT F*	AGGGCATCCGCAATTCAG
36	*Putative NAC domain containing protein 94 RT R*	ATGGCAGCTTCGGTTCCTT
37	*Fatty acly-CoA reductase RT F*	CTGCGGTGATCGGGTGTT
38	*Fatty acly-CoA reductase RT R*	GGCTGGACCCTCAGGATCTT
39	*S-Recptor kinase PK3-like RT F*	CCCCAGACAAGCAAACCAAT

40	*S-Recptor kinase PK3-like RT R*	GGTGACGGCAAGGAATGC
41	*Serine/threonine protein kinase RT F*	CAGGAATGTTCACCGTGGAGAT
42	*Serine/threonine protein kinase RT R*	CCGGTCCAGTTGCCAGTTT
43	*Chitinase IV RT F*	TGCAGCTGTCGTGGAACTAC
44	*Chitinase IV RT R*	CTTGAACGTTAACGCCTGGT
