# Supplementary figures and images for: Overexpression of HVA1 Enhances Drought and Heat Stress Tolerance in Triticum aestivum Doubled Haploid Plants
Source: Cells. 2022 Mar 7;11(5):912. doi: 10.3390/cells11050912 (PMC8909738; doi:10.3390/cells11050912)

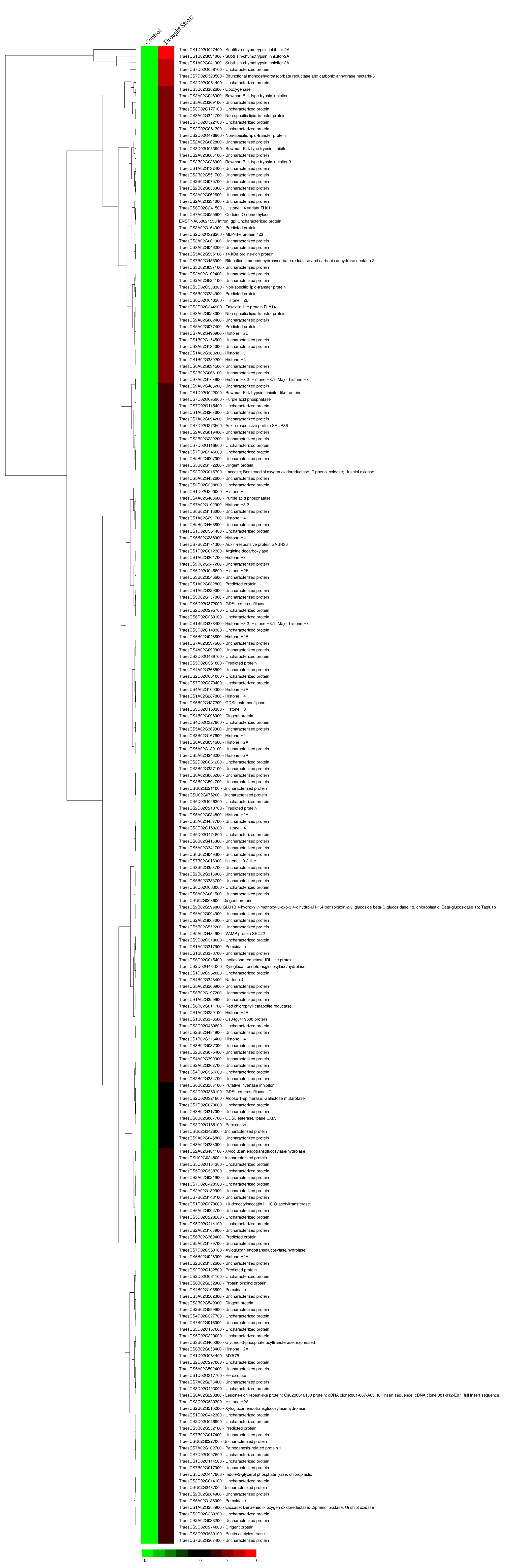

Supplement: Supplementary file 1 [file cells-11-00912-s001.zip › cells-1629540-supplementary/Figure S1.tif]

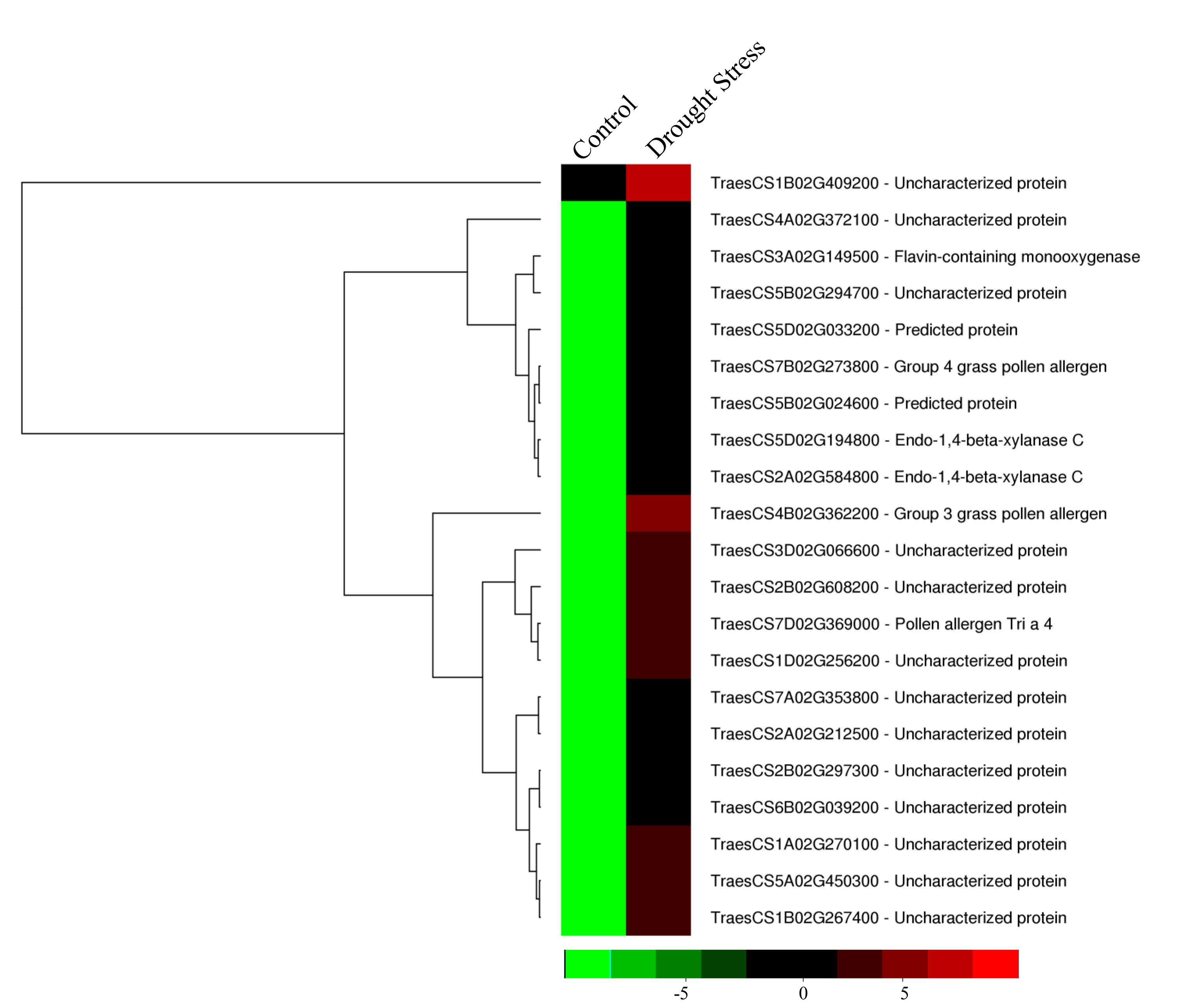

Supplement: Supplementary file 1 [file cells-11-00912-s001.zip › cells-1629540-supplementary/Figure S2.tif]

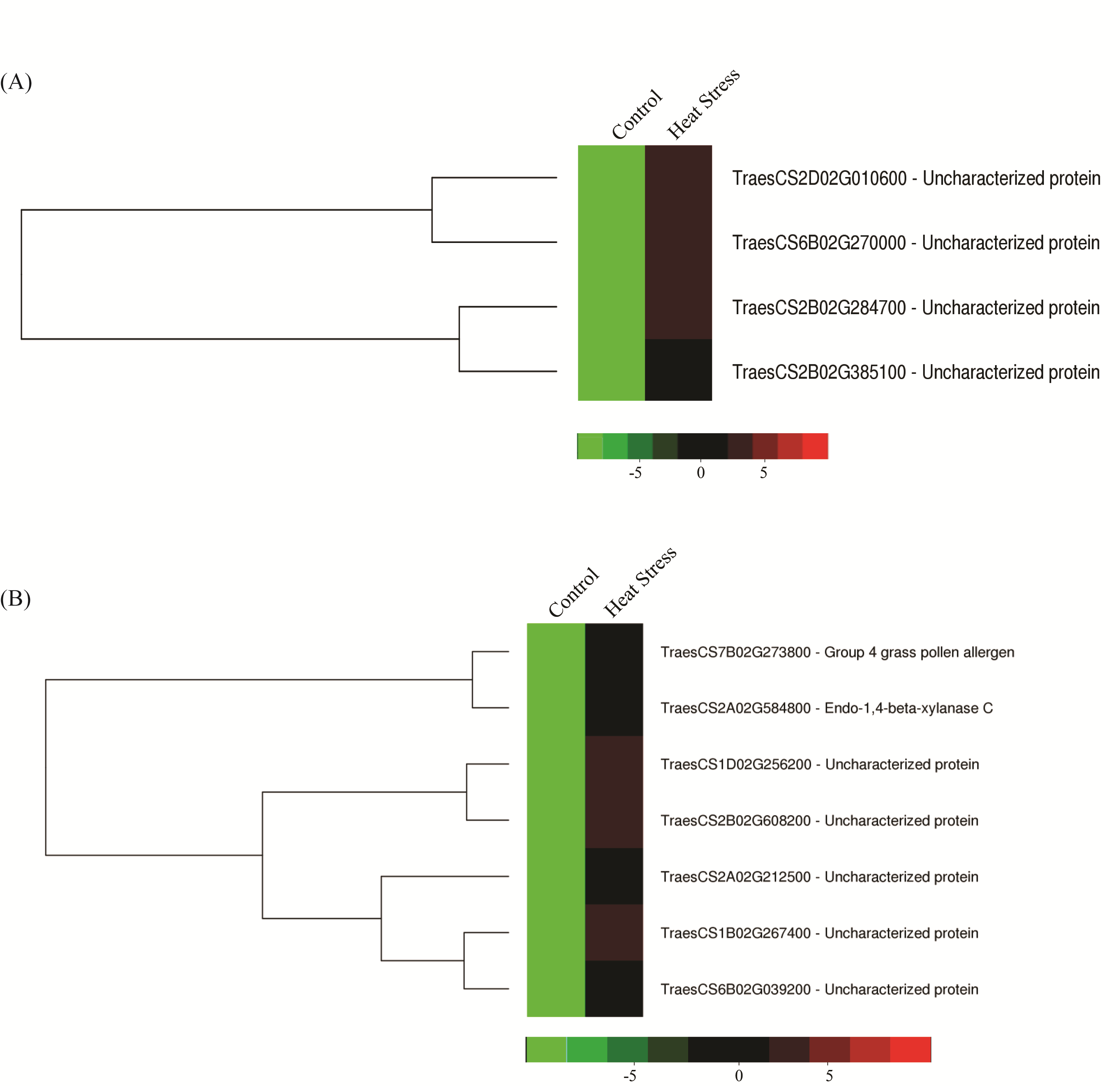

Supplement: Supplementary file 1 [file cells-11-00912-s001.zip › cells-1629540-supplementary/Figure S3.tif]
